# Approaches to teach evidence-based practice among health professionals: an overview of the existing evidence

**DOI:** 10.2147/AMEP.S134475

**Published:** 2017-07-07

**Authors:** Athina E Patelarou, Konstantinos G Kyriakoulis, Aliki A Stamou, Aggelos Laliotis, Dimitra Sifaki-Pistolla, Michail Matalliotakis, Emmanuel Prokopakis, Evridiki Patelarou

**Affiliations:** 1 Department of Anesthesiology, University Hospital of Heraklion, Crete; 2 Society of Junior Doctors; 3 Faculty of Medicine, National and Kapodistrian University of Athens, Athens, Greece; 4 Department of Oesophago-Gastric Surgery, Addenbrooke’s Hospital, Cambridge University Hospital NHS Foundation Trust, Cambridge, UK; 5 Clinic of Social and Family Medicine, School of Medicine, University of Crete, Crete; 6 Department of Obstetrics and Gynaecology, Venizeleio General Hospital, Heraklion; 7 Department of Otorhinolaryngology, School of Medicine, University of Crete, Crete, Greece; 8 Department of Family and Child Health, Florence Nightingale Faculty of Nursing and Midwifery, London, UK

**Keywords:** advanced clinical practice, health personnel, teaching strategies, nurses, physicians, lifelong education

## Abstract

Health care professionals’ adoption of evidence-based practice (EBP) remains limited, although most health care professionals are familiar with EBP and believe in its value. This systematic review aimed to bring together the best methods used to teach EBP to health professionals. The authors conducted a systematic search for the period 2005–2015 (an update of the search took place in October 2016) using PubMed interface (Medline). MeSH terms as well as free-text keywords were used. Studies were analyzed and evaluated by title and abstract. Those studies which fulfilled the inclusion criteria were assessed by full text. References of articles were also taken into consideration for identifying relevant studies not found through algorithm search. Twenty articles were found to be relevant. The majority of the studies were conducted among nurses (n=7) and physicians (n=6), and only a few among professionals from mixed disciplines (n=5). Two studies were conducted among chiropractors (n=1) and faculty members from a naturopathic and classical Chinese medicine institution (n=1). Researchers used a variety of different approaches, which varied with respect to duration and organization. We divided interventions into two categories. Single interventions included either a workshop, or a journal club, or a conference, or a lecture, or online learning tools, whereas multiple interventions included a combination of these approaches. An increase in EBP competencies and attitudes was reported in nine studies. Teaching methods for optimizing EBP among health professionals could become a robust standardized procedure of the medical educational curricula and lifelong learning of health care professionals.

## Introduction

The delivery of high-quality and safe patient care has been of utmost importance for all regulatory agencies. This has an impact on clinical decision-makers who encourage health care providers to incorporate evidence-based practices (EBPs) to provide high-quality clinical care. EBP integrates epidemiology, statistics, and research methodology into health care. The whole EBP process includes four steps: a) formulation of a focused research question, b) access relevant literature, c) critical appraisal of the validity of the existing research, and d) application of the findings to the decision-making.^1^

There is increasing evidence illustrating that health care professionals have a positive attitude towards EBP.^2^,^3^ Nonetheless, their understanding and skills related to EBP are inadequate.^2^–^4^ As a result, implementation of EBP has been challenging as only one in four health professionals provide care within an EBP framework.^5^,^6^ Today’s health care professionals have different levels of EBP knowledge and skills, depending on their education, experience, and own interest.^5^,^7^ Providing an organization of baseline information on EBP permits the development of educational initiatives and changes in order to enhance EBP incorporation into daily practice. As practices, beliefs, and skills of staff related to EBP may affect the success of initiatives to implement EBP, more research in these areas is valuable for assessing future implementation strategies.

This systematic review attempted to accumulate the existing knowledge and experience on how EBP strategies are taught over the last years with the aim to increase the awareness, knowledge, and positive attitudes and perceptions towards EBP of health professionals. The primary and main objective of this review was to explore and define the best approaches for teaching EBP to health professionals.

## Methods

Our research team has previously conducted a systematic review to summarize the present approaches for teaching EBP among health care students.^8^ As a further step, we decided to expand our search on the existing approaches to teach EBP to health care professionals, including medical doctors of any specialty and nurses, by incorporating a similar methodology approach.^8^ The review protocol of this review was based on the protocol developed for the review of health students already published elsewhere.^8^ Briefly, a protocol was developed according to the MOOSE Guidelines for Meta-analyses and Systematic Reviews of Observational Studies.^9^ Subsequently, a systematic search of Medline database was performed for the period 2005 to March 2015 (updated in October 2016). The core algorithm used was: ((“Physicians” OR “General Practitioners” OR “Nurses” OR “Nurse Midwives” OR “Health Personnel”) AND (“Models, Educational” OR “Education” OR “Health education” OR “Education, Nursing, Graduate” OR “Teaching”; “Curriculum” OR “Training” OR “Critical appraisal” OR “Workshops” OR “Journal clubs”) AND (“Evidence-Based Practice” OR “Evidence-Based Nursing” OR “Evidence-Based Dentistry” OR “Evidence-Based Emergency Medicine”)). The search was repeated for the EMBASE database. Hand-searching of additional eligible studies that met the inclusion criteria took place through screening the bibliographies of relevant studies/reviews. Same selection criteria were also applied in this review.^8^ Briefly, high-quality literature published in English over the last 10 years was included. Any type of educational intervention of a pre-/post-intervention style with a quantitative estimation of the effectiveness of the intervention was eligible for inclusion in this study. Literature screening (a three-stage approach-exclusion by reading the title, the abstract, and the full text) and extraction of the data were conducted by two reviewers independently. In cases of uncertainty, a discussion was held among the members of the team to reach a common consensus.

## Results

### Literature search

**Figure 1:**
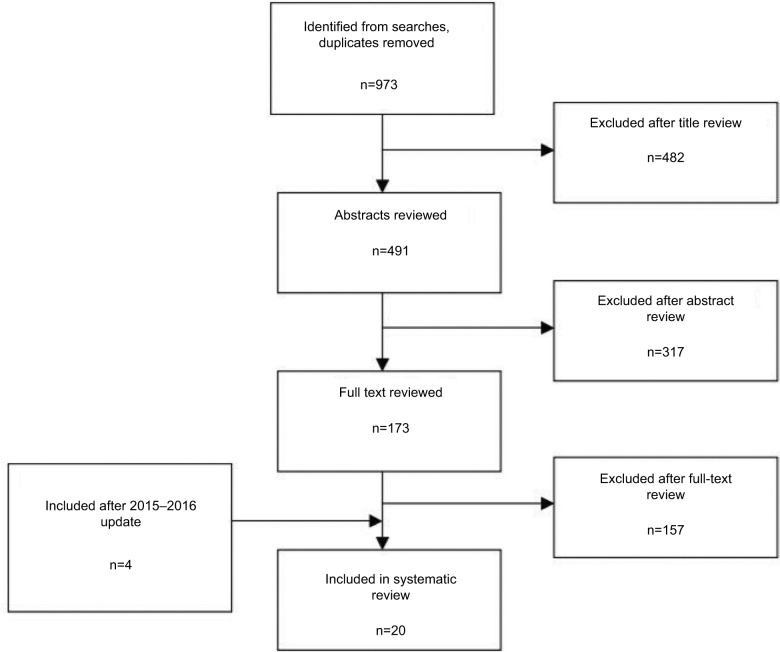
Flowchart for selection of systematic reviews of teaching strategies for evidence-based practice among health professionals.

A total of 973 records were retrieved through our search in Medline and Embase databases. After reading the articles and abstracts of the retrieved records, we selected 173 for further evaluation. Of these articles, 157 were excluded after reading the full text. Four articles were added following the updated search (October 2016). Finally, 20 studies were considered to be appropriate for answering our primary research question. Figure 1 shows the exact sequence and process of identification, selection, and exclusion of study in each step of the search.

### Study characteristics

Regarding the origin of each study, only one study was conducted in Europe (Spain)^10^, and 19 in countries outside Europe including the USA,^11^–^23^ Taiwan,^24^ Canada,^25^ Peru,^26^ Iran,^27^ Pakistan,^28^ and Israel.^29^ Twelve studies have been published since 2011.^10^,^11^,^14^,^15^,^18^,^20^–^26^ Of the 20 studies, 17 were pre–post uncontrolled trials,^11^,^12^,^14^–^20^,^22^–^26^,^28^,^29^ and three were controlled trials.^10^,^21^,^27^ In the studies, the effect of the intervention on the intervention group was compared to the attitudes of the nonintervention control group. The sample size varied from six to 609 health professionals. Participants of studies were mainly nurses (n=7)^10^–^13^,^19^,^20^,^22^ and physicians (n=6).^16^,^17^,^25^–^27^,^29^ The remaining studies sampled professionals from mixed disciplines (n=5),^15^,^18^,^23^,^24^,^28^ chiropractors (n=1),^21^ and faculty members from a naturopathic and classical Chinese medicine institution (n=1).^14^ Studies tested different educational approaches and interventions that varied in duration, design, and format (workshops, lectures, conferences, journal clubs, tutorials, online sessions, competitions) or a combination of these (multiple interventions, MIs). We classified studies as those including a single intervention (SI) (e.g. workshop, lecture, or online learning)^11^–^14^,^18^,^20^,^22^,^24^–^28^ and those including combination of different educational approaches and tools (multiple interventions).^10^,^16^,^17^,^19^,^21^,^23^,^29^ Moreover, the duration of the educational interventions varied from 2 hours to 2 years. Interventions covered different aspects of EBP including the formulation of a research question, existing sources of evidence, critical appraisal methodology, and interpretation of findings and implementation into practice. All but six studies reported using reliable and valid instruments to assess the effect of interventions on EBP attitudes, skills, and perceptions.^17^,^18^,^21^,^22^,^25^,^28^ Two studies used the Clinical Effectiveness and Evidence-Based Practice Questionnaire,^18^,^22^ one the Fresno Test, CAMS Test, and Questions by course chairs,^25^ one the EBASE Questionnaire,^21^ one the Berlin Questionnaire,^28^ and one the Fresno Test.^17^ The overall knowledge, beliefs, and skills of health care professionals related to EBP were most commonly explored among studies. Most of the studies evaluated the short-term effects of the intervention, and only three studies tested the longer-term effects: one 21 and 60 days,^10^ one 9 and 16 months,^21^ and one study 1 year post-intervention.^20^ The study characteristics are summarized in Table 1.

### Data synthesis

We synthesized the key findings of the eligible studies as follows. Nine studies showed an overall increase in all EBP domains (knowledge, skills, attitudes) following the intervention indicating a higher EBP competence, and only two studies examined the effectiveness of the intervention in improving the skill of database searching.

A study performed in the USA by Allen et al^11^ among 225 nurses evaluated the impact of a SI (web-based course) on EBP competences. Participants reported significant improvement in EBP knowledge following the completion of the course. Sprague et al^25^ performed a 2.5-day workshop consisting of interactive lectures and small group breakout sessions (SI) with 62 health professionals. EBP knowledge and research methodology of the participants was significantly improved (38.2%±21.3 vs 51.7%±19.1, *P*<0.001) after the course, while a major improvement was found in their overall knowledge regarding clinical research methodology (*P*<0.001). Another study performed by Sciarra^22^ in the USA offered five 2-hour sessions (SI) to 33 intensive care unit (ICU) nurses. A significant increase in EBP application, EBP attitudes, and EBP skill level (*P*<0.01) was also reported.

Zaidi et al^28^ organized a 14-hour workshop composed of seven sessions, covering basic evidence-based medicine (EBM) concepts – development of a research question, literature search, and appraisal of articles – in order to demonstrate that EBM training can be implemented in developing countries despite minimal resources. The Berlin Questionnaire was used to evaluate the results of the intervention. A significant change in the scores of the participants after completion of the workshop was reported (mean 4.7 vs 7.6, *P*<0.001).

Nicholson et al assessed the impact of a 1-year case-based EBM workshop organized for 28 clinical educators.^17^ The participants reported a significant improvement of the use of EBM resources and their overall EBM knowledge (both *P*<0.001). An uncontrolled study, conducted in the USA by Green among 10 academic podiatric physicians, presented the design, delivery, and evaluation of a national EBM program.^16^ The participants stated that after the 2-day seminar, they were able to search efficiently for the best evidence (*P*=0.005) and to critically appraise the report of a clinical study (*P*=0.004).

In another study, Allen et al organized a 20-hour course for faculty members in a faculty of naturopathic and classical Chinese medicine institution. Significant changes in research attitudes (4.4 vs 4.9, *P*<0.02), critical appraisal attitudes (3.4 vs 4, *P*<0.04), and self-appraised skills (3.6 vs 4.3, *P*<0.01) were reported from the participants.^11^

Wilson et al designed an MI which consisted of an 8-week journal club that combined in-person sessions and private online sessions in social media site.^23^ The 36 health care professionals who participated stated a significant increase in EBP use (1.2±0.7 vs 1.7±0.7) and behaviors (3.0±0.4 vs 3.2±0.4).

The latest study was a controlled trial which examined the impact of a 5-hour educational workshop (MI) on primary care doctors.^29^ The study reported a significant impact of the intervention on both utilizing EBM resources (*P*=0.001) and EBM knowledge (*P*=0.000).

A quasi-experimental study conducted in the USA evaluated the results of three trainings conducted among practitioners from community-based organizations.^15^ Participants were found to improve their skills on locating evidence-based resources (3.0 vs 4.7, *P*<0.001), narrowing search findings (3.0 vs 4.6, *P*<0.001), and determining strategies to collect primary data (3.17 vs 4.63, *P*<0.001). Weng et al organized an EBM contest and used PICO (problem/population, intervention, comparison and outcome) tool to determine participants’ skills in formulating an answerable question, performing a literature search, critically appraising the evidence, and implementing into clinical practice teams.^24^ A significant increase was observed in the EBP knowledge and skills of the participants (*P*<0.001). Moreover, a significant increase was observed in their access to literature databases.

Two studies compared the efficacy of different types of interventions. Hatmi et al compared the effectiveness of organizing conferences, including from small group discussions to traditional conferences, in improving EBP competency.^27^ The results proved conference combined with group discussion to be a more effective strategy for teaching EBP. Another randomized trial sampled 293 chiropractors, who were allocated to either an online EBP intervention group or a no-intervention control.^21^ The online intervention consisted of three courses and four booster lessons. Intervention group showed an improvement in their EBP attitudes (mean scores difference =6.2, *P*<0.001) and skills (mean scores difference =10.0, *P*<0.001) following the intervention compared to the nonintervention group.

Two studies were performed in developing countries.^26^,^28^ The first study from Pakistan is described above.^28^ The second study performed in Peru sampled 220 clinicians, who participated in an annual 3-day course with interactive lectures and interactive workshops.^26^ An improvement was observed in the self-reported competence (2.0 vs 3.0, *P*<0.001).

Seven studies sampled nurses.^10^–^13^,^19^,^20^,^22^ Two of them were described previously.^11^,^22^ A study performed in Spain^10^ included 109 nurses. A brief EBP course was organized for the intervention group. EBP attitude, knowledge and skills, and practice before the intervention, and at 21 and 60 days following the intervention were assessed. Knowledge and skills were significantly improved 21 and 60 days post-intervention (3.65 vs 3.61, 4.89 vs 4.07, 4.92 vs 4.3), whereas no significant difference was reported in EBP attitude and practice. Rutledge and Skelton designed an intervention that offered a program consisting of four 6-hour sessions of classroom/computer laboratory work during the summer period.^20^ Nurses reported an increase in comfort to search for the best research/evidence (baseline: 4.45, immediate post-class: 5.00, end of 1-year program: 6.00) and in overall comfort (4.11, 4.89, 5.56). In a study performed by Munroe et al, an MI was conducted, but the study did not observe statistically significant results concerning EBP attitudes, knowledge, and skills.^19^ Also, Dearholt et al conducted a 2-day workshop including computer laboratory and evidence appraisal group meetings.^13^ Following the course, participants reported that they were able to describe the steps used in the process of EBP, how to do a literature search, and how to identify the best sources of evidence. Finally, in a study performed in 2004, the enrolled nurses participated in an 18- to 24-month project and reported that the program helped them grow professionally and that they planned to use EBP in the future.^12^

Finally, one study performed in the USA assessed the impact of an online learning module on the attitudes of 609 participants.^18^ The study did not reach any statistically significant conclusion.

## Discussion

The use of research evidence in clinical decision-making is a core skill for health care practice. Promoting and implementing EBP and associated skills in clinical practice is essential for patient safety, quality of care, and carers’ satisfaction. Our findings showed that promoting and encouraging educational approaches to teach EBP is associated with improved delivery of clinical practice, in the area of EBP. In addition, health care professionals’ exposure to the methodology of critical appraisal and synthesis of research articles with the use of journal clubs and EBP strategies encourages the implementation of EBP in clinical practice. Our data also suggest that the provision of different options of teaching EBP relates to an increased engagement of professionals with different learning styles and preferences. This review provides evidence to leaders, decision-makers, and educators in clinical settings on the possible approaches and strategy to promote EBP attitudes, knowledge, and skills within the health care settings.

In order to be able to plan educational activities and strategies for promoting EBP in an organization, the first and essential step is to gather data on EBP practices, beliefs, and associated skills on an organizational level. The process of incorporating EBP into the culture of an organization is long and requires leadership support and resources. Leadership can influence this positive attitude by creating opportunities for practice, which will increase the knowledge and skills regarding EBP. Time, different learning styles, and unconventional work schedules are some of the barriers that organizations are requested to minimize in order to facilitate EBP implementation. Towards this direction, health care organizations need to develop and implement educational interventions which will focus on health care professionals and aim to encourage them to use EBP in their routine clinical practice.

The results of this review also have numerous implications as they can be used to develop future EBP training and interventions that can be implemented on a broader scale and therefore have larger impact. Existing strategies focus on improving EBP knowledge, formulating research questions with the use of PICO tool, searching electronic databases, and critically appraising the literature. An EBP educational intervention which will cover all these key areas and will adopt both online and face-to-face learning materials may result in improvements in the EBP knowledge and skills of professionals. Future educational interventions should also consider including the use of online group discussions and other interactive approaches in an effort to provide personalized feedback, collaborative learning, and educator–student interaction, all of which are known to facilitate adult learning.^30^–^32^

A limitation of the existing evidence is the heterogeneity of interventions that have been used and outcomes measured which did not allow for a meta-analysis to be conducted. More comprehensive studies are necessary to be done in order to answer key questions regarding the effectiveness of different approaches to teach EBP. In addition, future research should take into consideration the effectiveness of use and application of new technology and online direct and instant communication abilities in enhancing online interactive learning and opinion exchange. Finally, further research is essential in order to assess the long-term impact and sustainability of the educational interventions, their cost-effectiveness, and the role of enhancing EBP knowledge in improving health outcomes.

## Conclusion

Multi-thematic teaching strategies for optimizing EBP for health professionals should become a robust standardized procedure of the health profession’s educational curricula and lifelong learning of health care professionals. Among the different types of teaching strategies, the online programs seem to be effective and comprehensive. These findings conveyed the existing knowledge on teaching strategies and their effectiveness, while they could be utilized in order to enhance participation in future EBP programs. A better understanding of what motivates practitioners to engage in online educational activities is needed. Online educational intervention can provide an opportunity to enhance professionals’ overall motivation and capacity for EBP, but is not adequate to make important changes in their EBP behaviors. Future educational approaches using online learning might consider increasing staff support related to new technologies as technological challenges may deter some participants.

## Figures and Tables

**Table 1:** Summary of studies conducted among health professionals

Study	Main study characteristics	Intervention and instrument	Main findings		
Schneider et al^21^	USA, cross-sectional national survey, January 2013–September 2014	MI: online seminar and lessons on EBP during a 7-month period	Pre-intervention vs 9 and 16 months post-intervention mean scores		
	293 chiropractors – IG: 147 CG: 146	EBASE Questionnaire	Attitude 70.0 vs 69.1, 69.4 vs 65.3, 69.7 vs 73.1, *P*<0.01 Skills 49.2 vs 53.6, 56.4 vs 49.0, 53.2 vs 55.4, *P*<0.001 Use 41.4 vs 45.7, 40.5 vs 43.3, 42.0 vs 46.3, *P*<0.001		
Allen et al^11^	USA, Oncology Nursing Society in Pittsburgh, December 2009–November 2013	SI: “Developing Skills for Evidence-Based Practice” web course	Pre- vs post-intervention mean scores		
	225 nurses	Study questionnaire, EBPQ Test	EBP knowledge – 52.6±5.6 vs 87.6±3.8, *P*=0.00 EBPQ test – practice of EBP: 4.46±2.4; attitude towards EBP: 5.69±1.46; knowledge/skills associated with EBP: 5.46±0.61		
Ramos- Morcillo et al^10^	Spain, Nursing Council of Jaen, 2013	MI: comprehensive course on EBP competencies with both online and face-to-face sections	Pre-intervention vs 21 and 60 days post-intervention mean scores		
	109 nurses – IG: 54; CG: 55	Study questionnaire	Knowledge and skills: 3.65 vs 3.61, 4.89 vs 4.07, 4.92 vs 4.3, *P*<0.05 only for 21 and 60 days post-intervention Attitude: 5.88 vs 5.97, 6.05 vs 5.85, 5.85 vs 5.99, *P*>0.05 Practice: 3.56 vs 3.77, 4.14 vs 4.31, 4.72 vs 4.47, *P*>0.05		
Wilson et al^23^	USA, Magnet^®^-recognized hospital	MI: journal club (8-week duration) attended either face to face, or through online site, or with a combination of methods	Pre-intervention vs post-intervention mean scores		
	36 health care professionals (72% nurses)	Study questionnaire	EBP use: 1.2±0.7 vs 1.7±0.7 EBP behaviors: 3.0±0.4 vs 3.2±0.4 EBP ability: 4.0±0.8 vs 4.2±0.7, *P*>0.05		
Weng et al^24^	Taiwan, 39 medical centers, 2009 and 2011	SI: EBM competition using PICO queries	Pre- vs post-competition mean scores		
	358 health care professionals (physicians, nurses, pharmacists, and other)	Study questionnaire	Knowledge about EBM principles is adequate: 3.42±0.71 vs 3.77±0.72, *P*<0.001 Skills concerning literature searching are adequate: 3.70±0.64 vs 3.87±0.68, *P*<0.001 Skill concerning critical appraisal is adequate: 3.48±0.71 vs 3.80±0.72, *P*<0.001 Skills concerning EBM principles are adequate: 3.40±0.74 vs 3.76±0.71, *P*<0.001		
Sprague et al^25^	Canada, Department of Surgery and Department of Clinical Epidemiology and Biostatistics (McMaster University), 2009	SI: 2.5-day seminar including interactive lectures and small group sessions	Pre- vs post-intervention mean scores		
	Participants: 62 health professionals	Fresno Test, CAMS Test, Questions by course chairs	38.2% (SD 21.3) vs 51.7% (SD 19.1) Absolute increase: 13.5% Relative increase: 35.3%, *P*<0.001		
Escoffery et al^15^	USA, Rollins School of Public Health (Emory University), Spring 2009	SI: 3 trainings with 7 different sections (presentations, small group workshops, etc)	Pre- vs post-intervention mean scores		
	47 practitioners from community-based organizations	Study questionnaire	Ability to locate evidence-based resources: 3 vs 4.7, *P*<0.001 Filter search results to be more precise: 3 vs 4.6, *P*<0.001 Define methods for the collection strategy of primary data: 3.17 vs 4.63, *P*<0.001		
Mollon et al^18^	USA, Sharp Grossmont Hospital	SI: online learning module (via organization’s intranet system, accessibility for 2 months)	Pre- vs post-intervention mean scores		
	609 participants among clinical staff	Clinical Effectiveness and EBPQ	Practice of EBP: 4.51 vs 4.46, *P*=0.692 Attitude towards EBP: 5.22 vs 5.16, *P*=0.585 Knowledge/skills associated with EBP: 4.55 vs 4.58, *P*=0.741		
Rutledge and Skelton^20^	USA, St. Joseph Hospital, 2008	SI: 4 6-hour sessions during summer with classroom or PC courses	Pre-intervention, immediate post-intervention, and 1 year after intervention mean scores		
	11 nurses	Study questionnaire	Comfort to find the best evidence: 4.45, 5.00, 6.00 Overall comfort: 4.11, 4.89, 5.56 Skill to find research evidence: 2.72, 3.27, 2.25 Overall skills: 2.93, 3.41, 2.02		
Sciarra^22^	USA, Hospital in Northeastern United States, 2010	SI: 5 2-hour sessions	Pre- vs post-intervention mean scores		
	33 ICU nurses	Clinical Effectiveness and EBPQ	EBP application: 3.77 (SD 1.44) vs 5.43 (SD 1.29), *P*<0.01 EBP attitudes: 4.77 (SD 1.36) vs 5.70 (SD 1.14), *P*<0.01 EBP skill level: 4.89 (SD 1.02) vs 5.21 (SD 0.98), *P*<0.01 Total scores of EBPQ: 12.40 (SD 3.18) vs 16.35 (SD 2.87), *P*<0.01		
Tomatis et al^26^	Lima (Peru), 2005–2009	SI: annual 3-day course (interactive lectures/case-based workshops), each day: 2–3 interactive lectures (45 min each), after lecture an interactive workshop (45 min)	Pre- vs post-intervention mean scores		
	220 clinicians	Study questionnaire	Self-reported competence: 2 vs 3, *P*<0.001 Self-reported importance of EBP for basic practice: remained the same before and after intervention (5)		
Allen et al^14^	USA, National College of Natural Medicine, 2007	SI: 20-hour intensive week-long course “Principles of EBM for Complementary and Alternative Medicine (CAM) Professionals”	Pre- vs post-intervention mean scores		
	11 faculty members	Study questionnaire	Research attitudes: 4.4 vs 4.9, *P*<0.02 Critical appraisal attitudes: 3.4 vs 4, *P*<0.04 EBM attitudes: 3.7 vs 3.9, *P*<0.07 Skills self-appraisal: 3.6 vs 4.3, *P*<0.01 Knowledge test: 11 vs 14.2, *P*<0.02		
Hatmi et al^27^	Iran, Tehran University of Medical Sciences	SI: 12 2-day programs (6 hours per day)	Differences between IG and CG		
	170 members of the medical faculty divided into two groups – IG: 86; CG: 84	Conferences followed by small group discussions and group activities	Increase in knowledge score: IG 17.84, CG 10.35, *P*=0.001		
		Study questionnaire	Improvement in attitude score: IG 18.62, CG 6.10, *P*=0.001 Improvement in EBM-related skills: IG 40.29, CG 12.50, *P*=0.001		
Zaidi et al^28^	Pakistan, Shifa College of Medicine and Nursing, before 2009	SI: 14-hour workshop composed of 7 sessions, covering basic EBM concepts: development of question on clinical practice, search of the available literature, and critical appraisal of evidence	Pre- vs post-intervention mean scores		
	14 health care professionals of several faculties	Berlin Questionnaire	Berlin Questionnaire score: 4.7 (SD=2.3) vs 7.6 (SD=1.0), *P*<0.001		
Munroe et al^19^	USA (Illinois), rural hospital in a community with a state university, before 2008	Organizational supports to affect nursing personnel regarding EBP (consultative relationship with a nurse researcher, 3 workshops over 10 weeks and other interventions)	Pre- vs post-intervention mean scores		
	200 registered nurses	Study questionnaire	Knowledge: 1.31 vs 1.85, *P*=0.005 Skill: 1.44 vs 1.73 *P*>0.05 Attitude: 1.64 vs 1.82, *P*>0.05		
Dearholt et al^13^	USA, Johns Hopkins Hospital and The Johns Hopkins University School of Nursing, March/April 2008	SI: 2-day workshop (Day 1: introduction to EBP+2-hour computer laboratory; Day 2: mock evidence appraisal group meeting)		Post-intervention mean scores	
	Members of the central hospital nursing quality committees – Day 1: n=45; Day 2: n=31	Study questionnaires		Describe the steps used in the process of EBP: 4.5, SD=0.6 Describe how to do a literature/evidence search: 4.4, SD=0.7 Identify how to find the best sources of evidence online: 4.4, SD=0.63	
Shuval et al^29^	Israel, north and center district of a large health maintenance organization	MI: EBM educational 5-hour intervention with workshop		Pre- vs post-intervention mean scores	
	175 primary care doctors – IG: 101; CG: 74	Study questionnaire		Impact of the MI on utilizing EBP resources: 1.1/4 vs 1.4/4, *P*=0.001 Impact of the MI on EBP knowledge: 22.4% vs 40.8%, *P*=0.000	
Nicholson et al^17^	USA, University of California San Diego School of Medicine	SI: a CME course: 9 90-min workshops, every 4–6 weeks, for 1 year		Pre- vs post-intervention mean scores	
	26 clinician educators from 2 university hospitals	Fresno Test		Self-reported EBM knowledge: 2.6 vs 3.3, *P*<0.0005 Skill for evaluating the validity of a scientific article: 31.4% vs 71.6%, *P*<0.0005 Overall literature appraisal skill score: 57.9% vs 78.4%, *P*<0.0005	
Green^16^	USA, six of the seven accredited colleges of podiatric medicine in the USA, one podiatric medical residency program, and the Veterans Affairs podiatry service, 2001	SI: 2-day workshop divided into 8 sessions		Pre- vs post-intervention mean scores	
	10 academic podiatric physicians representing	Study questionnaire		Efficiently search for the best evidence answering my questions: 3.1 vs 4.1, *P*=0.005 Critically appraise the report of a clinical study: 2.8 vs 3.8, *P*=0.004 “Commitment-to-change” evaluation – 3 months survey: 25% fully implemented, 48% partially implemented, 26% failed to implement; 12 months survey: 32% fully implemented, 48% partially implemented, 20% failed to implement	
Cullen and Titler^12^	USA, University of Iowa Hospitals and Clinics	MI – 18–24 months project: (1) 12 meeting days (12 months): 3 classroom days (interactive lesson), 1 conference day, and 8 facilitated workdays; (2) meetings aiming project evaluation and integration into education		Post-intervention mean scores	
	6 nurses	Study questionnaire		The EBP Staff Nurse Internship program helped me to grow professionally: 4.8 The internship program has given me the knowledge I need to use EBP to answer clinical questions: 4.5 As a result of the EBP internship, I plan to use EBP in the future: 4.5	

**Abbreviations:** MI, multiple intervention; EBP, evidence-based practice; IG, intervention group; CG, control group; SI, single intervention; EBPQ, EBP Questionnaire; EBM, evidence-based medicine; CME, continuing medical education; PICO, problem/population, intervention, comparison and outcome.
